# Cerebral *Taenia crassiceps* larvae infection in a 71-year-old immunocompetent male

**DOI:** 10.1007/s15010-022-01912-w

**Published:** 2022-09-09

**Authors:** Niklas Floß, Sebastian Dolff, Andreas Junker, Tobias Blau, Laurel Rauschenbach, Ulrich Sure, Oliver Witzke, Dennis Tappe, Andreas Schönfeld

**Affiliations:** 1 Department of Infectious Diseases, West German Centre of Infectious Diseases, Essen University Hospital, University of Duisburg-Essen, Hufelandstraße 55, 45147 Essen, Germany; 2 Institute of Neuropathology, Essen University Hospital, University of Duisburg-Essen, Essen, Germany; 3 Department of Neurosurgery and Spine Surgery, Essen University Hospital, University of Duisburg-Essen, Essen, Germany; 4 Center for Translational Neuro- and Behavioral Sciences, C-TNBS, Essen University Hospital, University of Duisburg-Essen, Essen, Germany; 5 Bernhard Nocht Institute for Tropical Medicine, Hamburg, Germany

**Keywords:** Taenia crassiceps, Central nervous system, Larvae, Neurocysticercosis, Tapeworm

## Abstract

We report a rare case of a cerebral infection with *Taenia crassiceps* tapeworm larvae in an immunocompetent 71-year-old German male. Initially, an intracerebral malignoma was suspected after the patient experienced stroke-like symptoms. After surgery, helminth larvae, later identified as *T. crassiceps*, were detected. Identification on the species level was possible by specific PCR and sequencing. After complete surgical removal, the patient was treated with albendazole and dexamethasone for two weeks. No residual symptoms were reported up to date.

## Background

Neurocysticercosis is a neglected disease and a major public health problem especially in sub-Saharan Africa and Latin America. It often leads to epilepsy and is usually caused by larvae of *Taenia solium* [1, 2]. Rare cases of neurocysticercosis due to other *Taenia* species, such as *T. crassiceps,* have been reported in the northern hemisphere. *T. crassiceps* is a tapeworm related to *T. solium* and *T. saginata*.

Final hosts of *T. crassiceps* are carnivores like foxes, wolves and coyotes as well as dogs or cats. The prevalence in European cats and dogs is much lower (< 1%) than in foxes (7.8% in Switzerland, 24% in Germany, and 26.4% in Lithuania) [3, 4]. Rodents and rabbits are intermediate hosts where the asexual proliferation of the tapeworm takes place in their body cavities. In contrast to tapeworms like *T. solium* and *T. saginata*, *T. crassiceps* is able to proliferate asexually in the larval (cystic) stage in intermediate hosts: the cysticerci produce posterior buds, which in turn develop into cysticerci themselves, quickly affecting the health status of the natural intermediate host. The small animals are eaten as prey and the larvae develop to full size strobilar tapeworms in its predators’ intestines. The eggs are then passed on in feces and ingested, for example, by a rat. This way the cycle repeats itself [5]. Humans are a dead-end intermediate host for *T. crassiceps* in contrast to being a final host for other tapeworms (*T. saginata* and *T. solium*) [6]. Infection of humans, mostly involving muscles and subcutaneous tissue, is rare and often associated with a compromised immune system [7–12]. There is only one report of an infection of the central nervous system, also from Germany, involving the cerebellum in an elderly immunocompetent woman [13]. Here, we report a temporal lobe infection of an immunocompetent male adult.

## Case description

**Fig. 1:**
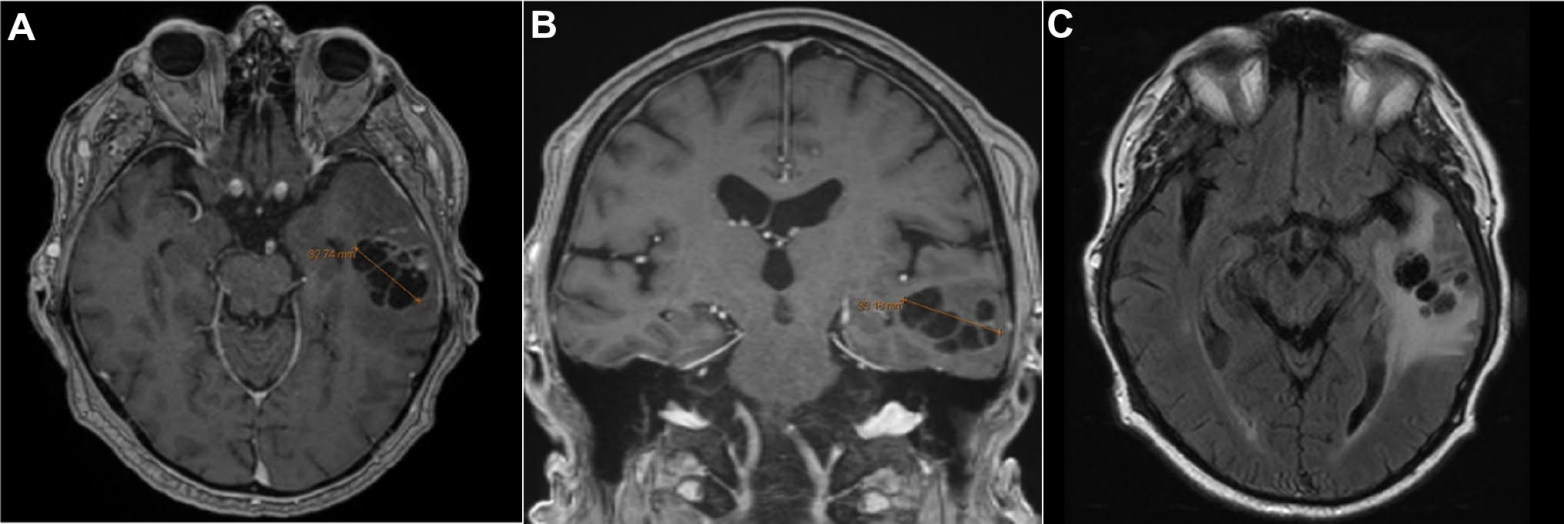
Magnetic resonance imaging (MRI) of an intracerebral, polycystic lesion of the left temporal lobe. The transverse view (**A**, T1-weighted image, contrast-enhanced) and the coronal view (**B**, T1-weighted image) show the lesion of approximately 30 × 30 mm in size in the left temporal lobe. The transverse view (**C**, Turbo Inversion Recovery Magnitude (TIRM) imaging) shows the perifocal edema caused by the lesion which was suspected to be a malignoma. No other intracerebral cysts could be detected. A slight midline shift can be observed. All images were acquired pre-surgery

A 71-year-old male patient was admitted to a regional hospital with symptoms suggesting an acute cerebrovascular event. He experienced trouble to speak fluently and revealed slightly impaired coordination while driving his car. Quick assessment including a computed tomography (CT) scan of the brain was performed. A stroke was ruled out but the first CT scan revealed a cystic tumor in the left temporal lobe. Cerebral magnetic resonance imaging (MRI) was performed to obtain high-resolution soft-tissue imaging (Fig. 1). It showed an intracerebral, polycystic, ring-enhancing and space-occupying lesion with perifocal edema and beginning midline shift to the contralateral side. The patient was transferred to the local neurosurgery department at our university hospital center and underwent microsurgical lesionectomy.

**Fig. 2:**
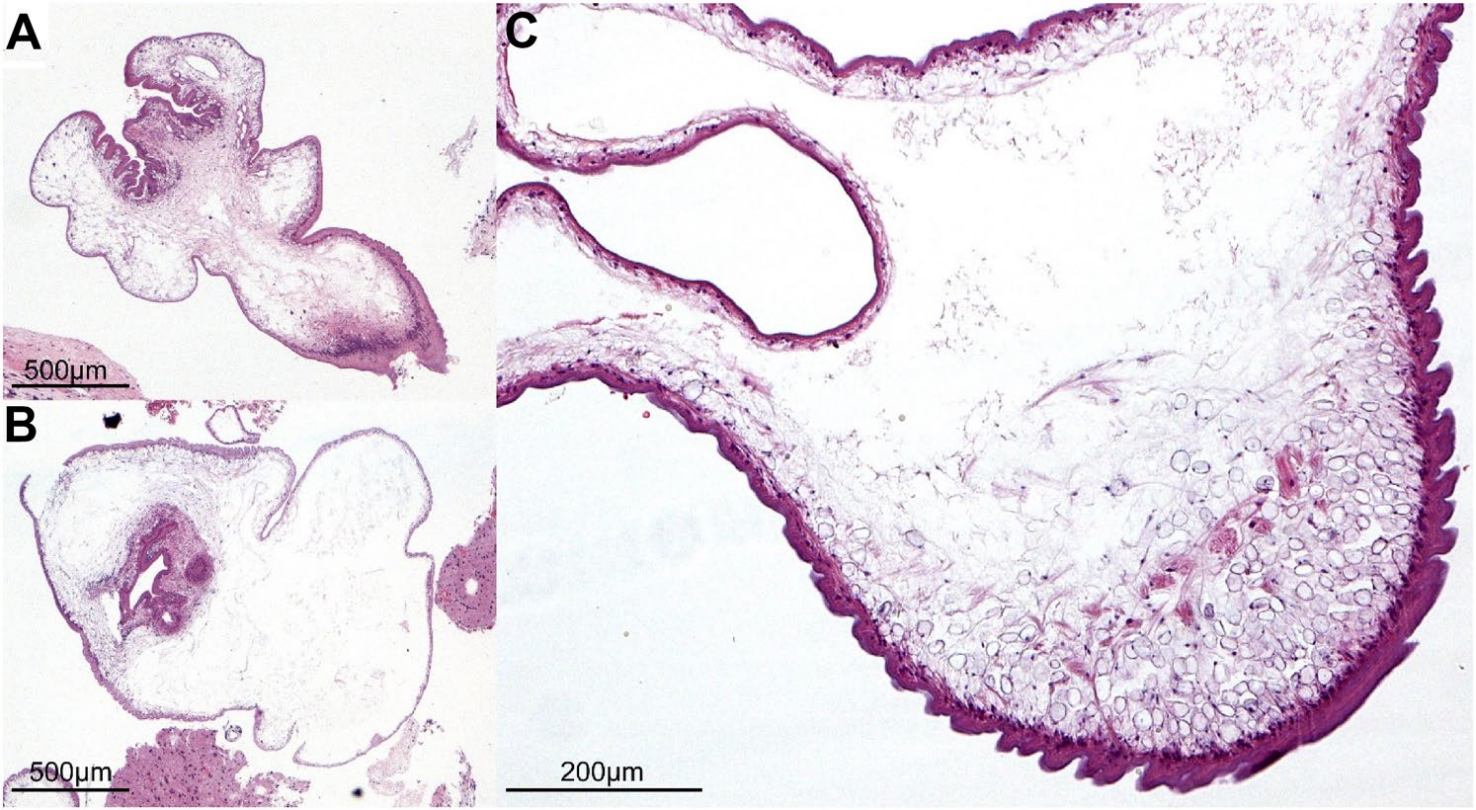
Histologic sections (hematoxylin and eosin stain) through Taenia crassiceps tapeworm larvae removed from the temporal lobe of a 71-year-old patient from western Germany. The images show an overview of the T. crassiceps larvae with a protruding protoscolex (**A**) and an invaginated protoscolex (**B**). Typical for T. crassiceps, multiple cysts can be seen on sections (**B**). Details of the tegument of the larvae are displayed in (**C**)

Surgery was performed without complications. Perioperatively, the 5-aminolevulinic acid method was applied to allow fluorescence-guided resection, but the lesion did not reveal 5-aminolevulinic acid uptake, which is unusual for intrinsic high-grade brain tumors like anaplastic astrocytoma (WHO CNS grade 3) and glioblastoma (WHO CNS grade 4) [14]. Nevertheless, haptic feedback of the lesion indicated a malignoma. Examination of the excised cystic tissue in the Institute of Neuropathology showed inflammation with infiltration of granulocytes, lymphocytes and plasma cells. Additionally, sections through multiple unidentified cystic helminth larvae were seen (Fig. 2). There was no sign of malignancy. The patient was transferred to our Department of Infectious Diseases for further diagnostics and treatment of the now established diagnosis of neurocysticercosis.

At the time of referral, the initial symptoms at first admission to the hospital had subsided. No anomalies in blood count (hemoglobin 15.5 g/dl, platelets 211/nl, eosinophils 0/nl) aside from mild leukocytosis (15/nl during medication with dexamethasone before and after brain surgery) could be detected. C-reactive protein and lactate dehydrogenase levels were not elevated. A CT scan of the thorax and abdomen was performed but no other cystic lesions or masses suspicious for an active tumorous disease were detected. Upon detailed clinical examination, the patient showed no other clinical signs of inflammatory disease or any palpable cysts. No lymphadenopathy was found. Existing comorbidities were type 2 diabetes mellitus (HbA1c 7.0%) with oral therapy (metformin), obesity (body mass index of 50 kg/m^2^), paroxysmal atrial fibrillation, controlled hypertension and psoriasis without immunosuppressive therapy. Serum antibody testing for *T. solium* infection and echinococcosis was negative (indirect hemagglutionation, enzyme-linked immunosorbent assay (ELISA), and immunoblot). No other serologic testing (e. g. for other cestodes) was conducted, and no cerebrospinal fluid was tested for antibodies against cestodes either. An HIV test was performed which was also negative. There was no history of any malignant disease.

Despite an apparent complete surgical resection of parasitic tissue, we started an anthelminthic therapy with albendazole (400 mg three times daily, which is the max. dose) and dexamethasone (4 mg three times daily for the first week of treatment followed by a tapered dose for another week) based on the WHO guidelines on treatment of *T. solium* neurocysticercosis [15]. The medication was tolerated well without adverse effects. No other alterations to the medication were made. Elevated blood sugar levels could be controlled with temporary insulin therapy.

**Fig. 3:**
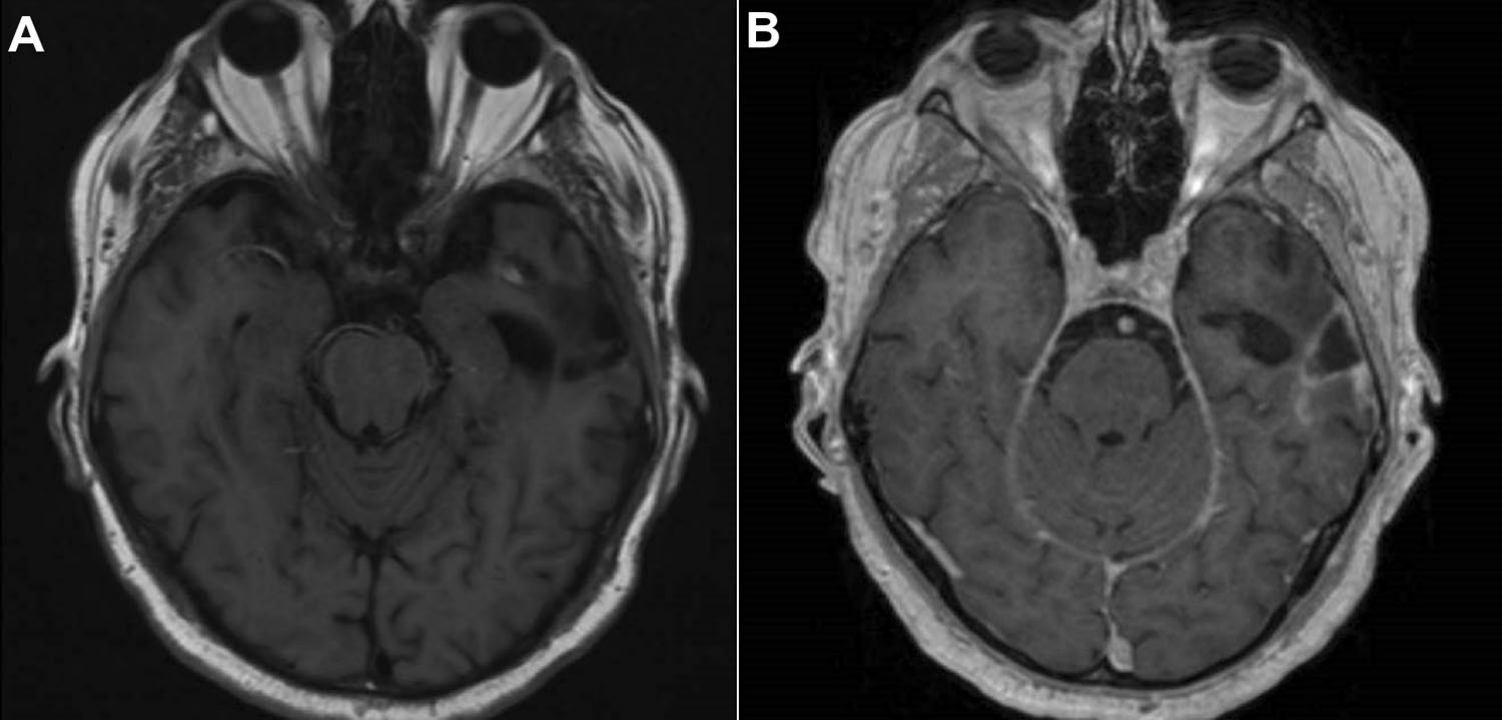
MRI at 3 months follow-up. The transverse view (**A**, T1 weighted image, not contrast enhanced; **B**, contrast-enhanced T1 weighted image) shows the area of the prior cystic lesion with a regressive perifocal edema

To identify the helminth species, a sample was sent to the Bernhard Nocht Institute for Tropical Medicine in Hamburg, Germany. Polymerase chain reaction (PCR) was performed from extracted DNA for cestode cytochrome oxidase subunit I gene. Sequencing of the 390 bp amplicon and Basic Local Alignment Search Tool (BLAST) [16] analysis revealed *T. crassiceps* as causative organism (100% homology with a *T. crassiceps* sequence from an infected zoo animal in the Czech Republic (KY321321)). The parasite’s cytochrome oxidase subunit I gene sequence was submitted to GenBank (OP103754). The medication was terminated after two weeks without any remaining symptoms at follow-up after three months. The MRI showed a regressive perifocal edema (Fig. 3).

It remained unclear where and when the patient was infected by *T. crassiceps* larvae. The patient had never owned a cat or a dog in his household, was not working as a farmer or hunter. Contacts to local animals with a high *T. crassiceps* prevalence (e. g. foxes in Germany) could not be remembered. The wife of the patient recalled the visit of a young family member having an unknown enteral “tapeworm” sometime in the past, however. Patient and wife live as retirees in western Germany.

## Discussion

Cysticercosis due to *T. crassiceps* larvae occurs by far more rarely than infection by the closely related *T. solium*. In contrast to infections with *T. solium*, humans do not serve as a final host for *T. crassiceps*. An increasing number of *T. crassiceps* infections in humans are reported [12, 13]. An underlying reason could be the increased availability of molecular diagnostic tools also for helminth infections, as exemplified by recent reports about *T. martis* as causative pathogen of a case of neurocysticercosis showing similar symptoms as our patient [17]. A study analyzing 12 samples of human cysticercosis cases discovered one infection by *T. crassiceps* und one more by *T. serialis* [18]. Other tapeworms like *T. multiceps* can also cause neurocysticercosis, clinically presenting as the larval tapeworm infections named above which can often only be distinguished by molecular diagnostics. In addition, the rising numbers of immunocompromised patients, for example after solid organ transplantation or HIV infection, can lead to opportunistic infections by cestodes, also by the recently proposed genus *Versteria* [19]. However, a compromised immune system does not seem to be a mandatory prerequisite for infections with *T. crassiceps*, as in a recent paper five out of 12 reported cases showed no signs of lacking immunocompetence [20] (an additional case of an immunocompetent patient was reported in 2014 from Germany [4]). Our patient was not under immunosuppressive therapy. We noted a severe obesity and type 2 diabetes which could have led to a compromised immune response or elevated susceptibility to infections, however [21].

Eosinophilia as typical sign of invasive helminthiasis is not a reliable finding in larval cestode infections including neurocysticercosis, as the immune system does not have to be activated by a single cyst [22]. Generally, in long-standing infections such as cysticercosis and echinococcosis, eosinophilia is usually absent. Moreover, serology for larval tapeworm infections has several limitations. First, owing to often few cysts, encapsulation by host fibrous tissue or localization in immuno-privileged sites such as in the central nervous system or in the eye, antibody production may be non-detectable [4, 23]. Second, if serological testing is positive, strong cross-reactions may be seen between different larval cestode infections [24].

We have chosen an additional albendazole therapy despite an assumed complete surgical resection of parasitic tissue, as neither imaging nor inspection of the surgical site intraoperatively can fully exclude any remaining small cysts of the multicystic lesion seen in our patient. Moreover, as larval *T.* crassiceps (which was identified as causative pathogen after the initiation of anthelminthic therapy), multiplies by asexual budding from a cellular progenitor zone of the cysts, we kept adhering to this therapeutic approach. Resection might have caused disruption of parasitic cysts and a possible dispersal of progenitor cells in the brain, another reason for an additional albendazole treatment.

Even if an extensive travel history can be obtained, it becomes clear that visits to tropical or developing countries is not a prerequisite for an infection with larval cestodes. *T. crassiceps* as an example in our report, *T. martis* as mentioned above, and *Echinococcus multilocularis* are all endemic in non-tropical, temperate geographical zones. Infections with *T. crassiceps* have to be considered in immunocompromised as well as immunocompetent patients. Definitive pathogen identification by molecular methods should always be attempted to put the patient’s infection in epidemiological context (different animal reservoirs for different tapeworms), for prognostic reasons (asexual multiplication of certain tapeworms) and to carefully decide best treatment options.
